# Cognitive stimulation and activities of daily living for individuals with mild-to-moderate dementia: A scoping review

**DOI:** 10.1177/03080226231156517

**Published:** 2023-03-15

**Authors:** Simone Ryan, Orla Brady

**Affiliations:** 1 Discipline of Occupational Therapy, University of Galway, Ireland; 2 HSE, Mental health Services, Longford/Westmeath, Ireland

**Keywords:** Activities of daily living, cognitive stimulation, dementia, multi-component intervention, occupational therapy, non-pharmacological approach

## Abstract

**Introduction:**

Dementia is a progressive syndrome that interferes with the individual’s ability to perform activities of daily living (ADL). Cognitive stimulation (CS) is a non-pharmacological approach aimed to mitigate the impact of dementia symptoms. While CS has been shown to provide benefits for cognition and quality of life, the evidence supporting its use in improving ADL outcomes is reduced. The aim of this review was to chart what is known from the literature about the use of CS in improving ADL outcomes.

**Method:**

A scoping review of the use of CS in improving ADL outcomes for individuals with mild-to-moderate dementia was conducted, following a scoping review methodological framework. Eight databases were searched, including all articles published up until June 2022.

**Findings:**

A three-step search strategy yielded 788 results. Following screening and review, 36 papers met the inclusion criteria for this review. Studies were charted and discussed in the areas of (1) cognitive stimulation therapy; (2) group CS programmes; (3) multi-component CS interventions; (4) individual CS programmes and (5) other types of CS.

**Conclusion:**

The review identified a range of CS programmes from across 13 countries worldwide. Multi-component CS interventions involving ADL-focused activities reported the most benefits for ADL outcomes.

## Introduction

Dementia, also referred to as major neurocognitive disorder (NCD), affects approximately 47 million people worldwide (Arvanitakis et al., 2019). It is a progressive syndrome characterised by deteriorating cognitive, social and behavioural functions that interfere with the individual’s ability to perform everyday activities at their previous capacity (American Psychiatric Association, 2013). Everyday functioning can be categorised into basic activities of daily living (BADLs) and instrumental activities of daily living (IADLs). IADLs, such as meal preparation and household maintenance, demand cognitive functions of a greater complexity than BADLS, and are therefore more likely to be affected in the early stages of dementia. Deficits in BADLs such as dressing or toileting are noted in the later stages of the disease (Sikkes et al., 2009). This functional decline is linked to reduced quality of life, increased care costs and increased carer burden (Laver et al., 2016). As of 2021, an estimated 64,888 individuals in Ireland live with the condition; a number that is predicted to rise to 98,946 by 2036 (Pierse et al., 2020). The estimated rise in individuals living with dementia has necessitated the development of feasible and effective interventions to slow the associated functional decline. While pharmacological therapies developed have shown moderate benefits for cognition and quality of life, non-pharmacological approaches are increasingly being used to help mitigate changes in cognition, mood and function (Justo-Henriques et al., 2022). Cognitive stimulation (CS) is one such non-pharmacological approach.

CS involves engagement in a range of activities and discussions aimed at general enhancement of cognitive and social functioning. This is achieved by the delivery of group or individual sessions in stimulating and rewarding social environments (Clare and Woods, 2004). Cognitive stimulation therapy (CST) is a brief group intervention based on these CS principles (Streater et al., 2016). The programme, designed for individuals with mild-to-moderate dementia, consists of 14 sessions of themed activities and discussions run over 7 weeks (Spector et al., 2011). A follow-up maintenance CST (MCST) programme run over 24 weeks is available, and individualised CST (iCST) has also been developed; however, the benefits of group CST have not yet been replicated through the individual format (Orrell et al., 2017).

Spector et al.’s (2003) initial randomised control trial (RCT) of CST involved 201 participants with mild-to-moderate dementia, with significant improvements in cognition and quality of life observed in the treatment group. Positive trends in communication were also shown. These results were supported by a Cochrane review of 15 RCTs with a total of 718 participants, where consistent benefits were demonstrated for cognition and secondary benefits noted for quality of life, communication and social interaction (Woods et al., 2012). While it is assumed that cognitive gains will generalise to daily life functioning, there is a notable lack of evidence supporting the benefits of CS for maintaining performance in daily activities. Woods et al.’s (2012) systematic review of CS found no statistically significant effect of CS on activities of daily living (ADLs). In contrast, Garrido-Pedrosa et al.’s (2017) systematic review of cognitive-focused interventions for dementia provided evidence of benefits of CS groups in maintaining performance in ADL. However, Dolan et al.’s (2022) more recent pilot study comparing CST to Sonas, a non-pharmacological, multi-sensory stimulation treatment approach, disputed these findings, as no significant between-group differences were found for ADL performance.

CST is often delivered by occupational therapists, a profession where occupational performance and engagement in ADLs are central to intervention. The limited evidence available to support the use of CS for ADLs calls into question the widespread use of CST by occupational therapists with the dementia population. However, a pilot RCT (*N* = 58) investigating the effects of an occupational therapy CS programme on ADL skills reported significant improvements in the ADLs of feeding, dressing, continence and stair mobility (Jiménez Palomares et al., 2021). These results are promising, indicating more research is needed to investigate whether CS is more effective for occupational therapy goals when used as part of an ADL-focused, multi-faceted intervention (Dolan et al., 2022).

A scoping review of the literature is therefore warranted to identify the evidence available for the use of CS in ADL interventions for individuals with mild-to-moderate dementia. This review will align with a pilot CS ADL group intervention currently underway at the time of writing this review. This pilot group for people with mild-to-moderate dementia will incorporate activities of daily living into a typical CS program. The results obtained from the review and the pilot group will be used to inform a research proposal for a larger trial of a CS ADL group intervention for individuals living with mild-to-moderate dementia. A preliminary search of the Cochrane Database of Systematic Reviews, JBI Evidence Synthesis, CINAHL and PubMed was conducted on the 13th of June 2022, and no current or underway systematic or scoping reviews on the topic were identified. The rationale for not including research with individuals with severe cognitive impairment is that the nature of the intervention for those individuals would be different and most CS interventions are not applicable for those with severe cognitive impairment (Spector et al., 2003).

The following research question was thus formulated for this scoping review: What is known from the existing literature on the use of CS in improving ADL outcomes for individuals with mild to moderate dementia?

## Methodology

A scoping review methodology was chosen due to the exploratory objectives of this research. This review aims to map the range of evidence available for the use of CS in improving ADL outcomes, to identify key characteristics of CS ADL interventions, and to analyse any existing knowledge gaps. Arksey and O’Malley’s (2005) scoping review framework, as amended by Levac et al. (2010) and Peters et al. (2020), was followed. Best practice reporting criteria as per the PRISMA-ScR guidelines were also followed (Tricco et al., 2018); however, a protocol was not registered and published due to time constraints when conducting this review (Table 1). The following stages were used to conduct the review.

**Table 1.** Preferred Reporting Items for Systematic Reviews and Meta-Analyses extension for Scoping Reviews (PRISMA-ScR) checklist.

Section	Item	PRISMA-ScR checklist item	Reported on Page #
Title			
Title	1	Identify the report as a scoping review.	1
Abstract			
Structured summary	2	Provide a structured summary that includes (as applicable): background, objectives, eligibility criteria, sources of evidence, charting methods, results and conclusions that relate to the review questions and objectives.	1
Introduction			
Rationale	3	Describe the rationale for the review in the context of what is already known. Explain why the review questions/objectives lend themselves to a scoping review approach.	2
Objectives	4	Provide an explicit statement of the questions and objectives being addressed with reference to their key elements (e.g. population or participants, concepts and context) or other relevant key elements used to conceptualise the review questions and/or objectives.	2
Methods			
Protocol and registration	5	Indicate whether a review protocol exists; state if and where it can be accessed (e.g. a Web address); and if available, provide registration information, including the registration number.	N/A
Eligibility criteria	6	Specify characteristics of the sources of evidence used as eligibility criteria (e.g. years considered, language and publication status), and provide a rationale.	2,4
Information sources	7	Describe all information sources in the search (e.g. databases with dates of coverage and contact with authors to identify additional sources), as well as the date the most recent search was executed.	2
Search	8	Present the full electronic search strategy for at least 1 database, including any limits used, such that it could be repeated.	Appendix A1
Selection of sources of evidence	9	State the process for selecting sources of evidence (i.e. screening and eligibility) included in the scoping review.	2
Data charting process	10	Describe the methods of charting data from the included sources of evidence (e.g. calibrated forms or forms that have been tested by the team before their use and whether data charting was done independently or in duplicate) and any processes for obtaining and confirming data from investigators.	4
Data items	11	List and define all variables for which data were sought and any assumptions and simplifications made.	Table 2
Critical appraisal of individual sources of evidence	12	If done, provide a rationale for conducting a critical appraisal of included sources of evidence; describe the methods used and how this information was used in any data synthesis (if appropriate).	N/A
Synthesis of results	13	Describe the methods of handling and summarising the data that were charted.	4
Results			
Selection of sources of evidence	14	Give numbers of sources of evidence screened, assessed for eligibility, and included in the review, with reasons for exclusions at each stage, ideally using a flow diagram.	Figure 1
Characteristics of sources of evidence	15	For each source of evidence, present characteristics for which data were charted and provide the citations.	Table 2
Critical appraisal within sources of evidence	16	If done, present data on critical appraisal of included sources of evidence (see item 12).	N/A
Results of individual sources of evidence	17	For each included source of evidence, present the relevant data that were charted that relate to the review questions and objectives.	Table 2
Synthesis of results	18	Summarise and/or present the charting results as they relate to the review questions and objectives.	4-15
Discussion			
Summary of evidence	19	Summarise the main results (including an overview of concepts, themes and types of evidence available), link to the review questions and objectives, and consider the relevance to key groups.	15
Limitations	20	Discuss the limitations of the scoping review process.	15-16
Conclusions	21	Provide a general interpretation of the results with respect to the review questions and objectives, as well as potential implications and/or next steps.	16
Funding	22	Describe sources of funding for the included sources of evidence, as well as sources of funding for the scoping review. Describe the role of the funders of the scoping review.	16

Source: Tricco et al. (2018).

### Stage 1: Identifying the research question

A clear research question was formulated to identify the focus of the review. This question was framed using the Population, Concept and Context (PCC; Peters et al. 2020). The question was based on gaps in knowledge noted previously by Dolan et al. (2022).

### Stage 2: Identifying relevant studies

A three-step search strategy was utilised for this review. An initial limited search of PubMed and CINAHL was completed to become familiar with the literature. Text words in the title and abstract of relevant studies and article index words were analysed to identify keywords to be used in the next stage of the search.

A second search using all identified keywords and terms was then completed across the following eight electronic databases: Medline, CINAHL, PubMed, the Cochrane Library, Scopus, PsycINFO, OTseeker and Embase. These searches were completed on the 15th and 16th of June 2022. A broad variety of terms were used to describe each concept as terms are used interchangeably across the literature. MeSH terms, Boolean logic and truncation were used where appropriate and adapted accordingly for each database. Full search strategies are presented in sequence in Appendix A1. The search limits of ‘English language’ and ‘full-text’ were set where applicable due to the limited translation facilities of the authors and time pressures of the screening process.

Reference lists of relevant articles identified from the first two stages of the review were then hand-searched. Grey literature and relevant journals were also searched for additional articles.

### Stage 3: Study selection

Search results were imported into Rayyan, a web application for systematic reviews that was used throughout the study selection stage (Ouzzani et al., 2016). Through Rayyan, duplicates were removed, and screening of articles was completed in a two-stage process; titles and abstracts were screened first followed by the screening of full-text articles. Articles were included if they were full-text, published in the English language and included participants with mild-to-moderate dementia or major NCD. Study populations that reviewed both mild-to-moderate dementia participants alongside individuals with severe dementia or mild cognitive impairment (MCI) were included, however results were interpreted with caution. Included articles must involve a programme of CS and evaluate ADLs (or associated terminology) as an outcome. Studies were excluded if they did not include participants with an established diagnosis of dementia or major NCD, or only included participants with dual diagnoses, for example, intellectual disability and dementia. If an article’s full text was not available after appropriate steps to retrieve it, it was excluded. Inclusion and exclusion criteria were refined and adjusted iteratively by the authors as familiarity with the literature was developed.

At the stage of full-text screening, a decision was made to only include primary research papers, including but not limited to, randomised controlled trials (RCTs), quasi-experimental studies, case control, analytical cross-sectional studies, cohort and qualitative studies. Systematic reviews, meta-analyses and literature reviews were excluded.

### Stage 4: Charting the data

A data charting form was initially drafted based on the template provided by the JBI methodology guidance for scoping reviews (Aromataris and Munn, 2020), with updates and refinements made continually to ensure alignment with review objectives.

### Stage 5: Collating, summarising and reporting results

As recommended by Levac et al. (2010), a descriptive numerical analysis was completed to gather information regarding year of publication, sample characteristics and research methods used. Characteristics of intervention and ADL outcomes were subsequently analysed. This data was then collated and summarised in Table 2, followed by a thematic summary of results according to intervention type.

**Table 2.** Charting of included studies.

Author(s)	Study design	Level of evidence^a^	Sample	Intervention approach	Frequency, intensity, duration	Comparator	Relevant outcome measure	Results
Alvares-Pereira et al. (2021)	Single-blind, multi-centre RCT	Level I	Portugal, *N* *=* 112, 62.9% residential care, 37.1% live at home, 23.8% day-centre attendees. Age range = 59–98 years, 86.7% women.	CST-Portuguese	14 sessions over 7 weeks, twice weekly, 45–60-minute sessions.	Unstructured activities control group	Clifton Assessment Procedures for the Elderly-Behaviour Rating Scale (CAPE-BRS)	CST group showed significant improvements in functionality; bathing, mobility, dressing, combing, going out, urinary incontinence (*p* = 0.008).
Alves et al. (2014)	RCT	Level I	Portugal, *N* *=* 17 from day care and long-term care centres.	Standard CS programme (discussions and reminiscence of songs, games, events, gardening; identification and categorisation of objects, photos; memory exercises)	17 sessions, 1 hour each over 1.5 months. 3 sessions per week, 2 sessions in the final week.	Brief CS programme of 11 sessions for 1 month	IADL Scale	No significant between-group difference (*p* = 0.07).
Ávila et al. (2018)	Multiple-baseline, intrasubject design	Level II	Spain, *N* *=* 21, community dwelling older adults with mild AD, mean age = 78.6 years.	Home-based, high intensity multicomponent occupational therapy intervention, consisting of CS, meaningful activities, psychomotor and sensory skills activation, home medication, caregiver counselling and ADL training	12 weeks, twice weekly, 90-minute sessions. After 1.5-month withdrawal, intervention replicated for 8 weeks. Overall, 6.5 months.	N/A	Barthel Index	Statistically significant change in total score between T1 and T4 assessment.
Breuil et al. (1994)	RCT	Level I	France, *N* *=* 56, community dwelling adults, mild-to-moderate dementia	CS programme (pen-and-paper tasks, discussion, identification, categorisation, association)	10 sessions, 1-hour each over 5 weeks.	No treatment	ECA (*échelle comportemenale adaptive*) (behaviour adaptation scale)	76% maximum scores at T1, 78% maximum score at T6; ADL scale not suited for study.
Capotosto et al. (2017)	Single-blind, multi-centre RCT	Level I	Italy, *N* = 39 from 2 residential homes	CST adapted to Italian context	14 sessions, twice a week over 7 weeks	Active control group of educational activities; reading newspapers/stories, creative activities.	Disability assessment for dementia (DAD)	Post-test DAD scores lower in CST group, however no significant effect.
Chapman et al. (2004)	RCT	Level I	US, *N* *=* 41, community-dwelling, mild to moderate Alzheimer’s disease (AD), age range = 54–91 years, 54% female, 46% male.	Donepezil plus CS (conversational group interaction; discussions and reminiscence of current events, politics, news, personal activities, Alzheimer’s, childhood, family, marriage, hobbies, work)	8 weeks, 5-hour sessions once a week	Donepezil-only group	Texas functional living scale (TFLS)	No significant effect for CS group, however less functional decline in CS group compared to donepezil-only group.
Chang et al. (2021)	Pre- and post-test experimental study design with two randomly assigned groups	Level II	Taiwan, *N* *=* 23, private day care centres	Multicomponent CS programme (MCCSP) (structured cognitive and leisure activity programme)	At least 1 hour per session, 3 times per week for 12 weeks	Usual care	CAPE-BRS; Refined ADL assessment scale	CAPE-BRS demonstrated statistically significant between-group differences (*p* = 0.017), however ADL score was not significant (p = 0.430)
Coen et al. (2011)	RCT	Level I	Ireland, *N* *=* 27, mild-to-moderate dementia, long-term care residents, education most exclusively to primary school level.	CST and usual care	14 sessions twice weekly over 7 weeks, 45 minutes per session	Usual care (e.g., Sonas, bingo, music, art)	CAPE-BRS	No significant changes in function as measured by the CAPE-BRS (*p* = 0.45)
Cruz et al. (2015)	Case series	Level V	Brazil, *N* *=* 5, aged over 71, 4–8 years of schooling, AD, community-dwelling.	Individual CS	1.5-hour sessions delivered weekly, 12 sessions total	N/A	Katz ADL scale, Lawton IADL scale	No change on Katz ADL scale. Lawton IADL scale score increased for three participants.
Dolan et al. (2022)	Single-blind prospective controlled trial	Level II	Ireland, *N* *=* 28, inpatient and community settings. Mean age = 80.29 years	CST	Twice a week, 14 sessions, 7 weeks.	Sonas group	Alzheimer’s Disease Cooperative Study ADL Scale (ADCS-ADL)	No significant between-group differences (*p* = 1.000).
D’Onofrio et al. (2015)	Pilot RCT	Level I	Italy, *N* *=* 90, 46.7% men, 53.3% women, mean age = 78.19 years, AD, outpatient.	Rivastigmine transdermal patch (RTP) (dose 9.5 mg/24 hours) with individual CS (adapted for cognitive level)	90-minute sessions, once a week for two cycles (each cycle lasting 2 months followed by a 2 month stop)	RTP only	Katz Activities of Daily Living (ADL) Scale and Lawton IADL Scale	Significant between-group differences measured by Katz ADL Scale (*p* = 0.001), and Lawton IADL Scale (*p* < 0.0001).
D’Onofrio et al. (2016)	Open RCT	Level I	Italy, *N* *=* 40, AD and vascular dementia (VaD), 9 men, 31 women, mean age = 84.56 years, institutionalised participants.	Individual training sessions with CS (adapted for cognitive level)	90-minute sessions, once per week for four cycles (each cycle lasting 2 months followed by a 1 month stop)	Usual care	Katz ADL Scale and Lawton IADL Scale	Significant differences in CS group in IADL (*p* < 0.0001) after 1-year follow-up.
Farina et al. (2006)	Quasi-experimental study	Level II	Italy, *N* *=* 32, community-dwelling participants divided into groups of 4	‘Global stimulation’ group (different recreational activities, conversation, singing, dancing, games, art)	15 sessions total for 6 weeks, 3 hours each.	Cognitive-oriented training (procedural memory training on ADLs and neuropsychological rehabilitation of residual cognitive functions)	Functional living skills assessment (FLSA)	FLSA improvement in global stimulation group post-treatment however results lost at follow-up.
Folkerts et al. (2018)	Pilot RCT of a crossover design	Level I	Netherlands, *N* *=* 12, Parkinson’s disease dementia (PDD), long-term care	NEUROvitalis senseful (structured CS programme) for PDD	8 weeks, 60-minute sessions twice a week.	Usual care including sport, music, art.	Barthel Index	Significant deterioration shown for Bathel Index (*p* = 0.014) for CS group
Graessel et al. (2011)	Randomised, controlled, single-blind longitudinal study	Level I	Germany, *N* *=* 61, long-term care.	MAKS	6 days a week for 12 months, 2 hours per session	Usual care	E-ADL	E-ADL scores remained stable for MAKS group at 12 months (*d* = 0.69), control group E-ADL scores decreased.
Ibarria et al. (2016)	Descriptive observation study	Level III	Spain, *N* *=* 206, mild to moderate AD Mean age = 75.88 years. Community-dwelling participants attending day care centre.	Integrated psychostimulation programme (IPP) combined with daily dose of AChEIs and/or memantine.	5 days a week, 10 am-6 pm. Some participants completed only three mornings per week	N/A	Rapid disability rating scale (RDRS-2)	Statistically significant worsening on ADL functionality (*p* < 0.001) however changes were not clinically significant
Jiménez Palomares et al. (2021)	Double-blind randomised clinical controlled trial	Level I	Spain, *N* *=* 58, institutionalised older adults with dementia/major neurocognitive disorder.	Occupational therapy programme based on the training of ADL through CS, delivered 1:1		Conventional occupational therapy intervention for the management of ADL deficits	Barthel Index	Significant improvement in OT CS group in ADLs post-intervention (*p* = 0.006) Improvement was not maintained at follow-up.
Justo-Henriques et al. (2022)	Single-blind, parallel two arm RCT	Level I	Portugal, *N* *=* 59, non-institutionalised participants with minor and major neurocognitive disorder, 50.8% AD, 30.5% VaD, 8.5% frontotemporal dementia, 19.2% other	Individual, home-based, CS programme inspired by iCST	45-minute sessions weekly over 23 weeks. Midpoint evaluation followed by another 24 weeks.	Usual care as provided by caregiver or day programme; activities based on socialisation or exercise.	Lawton and Brody index (LBI)	Group x time significant interaction found for LBI (*p* = 0.018), however no significant between-group difference.
Leroi et al. (2019)	Single-blind, randomised, controlled exploratory pilot trial	Level I	UK, *N* *=* 76, 21% female, 40 with PDD, 21 with dementia with Lewy bodies, 15 with Parkinson’s disease MCI. Non-institutionalised participants.	CST-PD; individualised care-partner delivered home-based CST sessions	30-minute sessions, two-to-three times per week, 12 weeks	Usual care including pharmacological and non-pharmacological treatment	The pill questionnaire	No significant difference between groups (*p* = 0.435)
Luttenberger et al. (2012a)	Randomised, controlled, single-blind, longitudinal study	Level I	Germany, *N* *=* 139 nursing home residents with degenerative dementia, mean age= 85, 83% women	MAKS and usual care	6 days a week for 6 months, 2-hour sessions.	Usual care including memory training, physical exercise, falls prevention, cooking groups, occupational therapy	Nurses’ Observation Scale of Geriatric Patients (NOSGER); Barthel Index	NOSGER ADL and IADL subscales showed significant difference in MAKS group (*p* = 0.05; *p* = 0.013); No significant changes observed in Barthel I (*p* = 0.49)
Luttenberger et al. (2012b)	Randomised, controlled, single-blind longitudinal trial	Level I	Germany, *N* *=* 52. MMSE average score=15, 83% female, average age = 84 years. Nursing home residents.	MAKS: multicomponent group therapy consisting of motor stimulation, ADLs, CS, and a spiritual element.	2-hour sessions run 6 days a week for 12 months	Usual care	Erlangen test of ADL (E-ADL)	MAKS group ADL abilities remained stable during therapy however deteriorated significantly between end of therapy and follow-up. Control group ADL abilities decreased significantly during and after therapy.
Marinho et al. (2021)	Single-blind RCT	Level I	Brazil, *N* *=* 47, mild-to-moderate dementia, outpatient	CST adapted to Brazilian context	14 sessions over 7 weeks, two sessions per week run on the same day for feasibility. 45 minutes per session.	Usual care combined with regular visits to a geriatric psychiatrist and AChEI prescription.	ADCS-ADL scale	Significant time × group interaction (*p* = 0.039). Medium effect size found for improvements in functional ability.
Muñiz et al. (2015)	RCT	Level I	Spain, *N* *=* 84, non-institutionalised with mild-to-moderate AD and MCI	Cognitive-motor stimulation intervention (CMSI) (individual cognitive exercises, group cognitive exercises, ADL training, psychomotor therapy/workshops)	3.5-hour sessions twice weekly over 3 years	Usual care	Functional Activities Questionnaire (FAQ)	Both groups showed progressive deterioration in functional variables, however group x time effect found the evolution in ADL and IADL favourable for CMSI group.
Olazarán et al. (2004)	RCT	Level I	Spain, *N* *=* 84, including MCI, mild AD and moderate AD, community-dwelling adults, all receiving AChEIs.	Cognitive-motor stimulation programme plus psychosocial support	Twice weekly sessions, 3.5 hours per session for 12 months.	Psychosocial support only.	FAQ	No significant improvement in FAQ between groups (*p* = 0.422)
Oliveira et al. (2021)	Open-label pilot RCT	Level I	Portugal, *N* *=* 17, mean age = 83 years.	Computerised CS programme with non-immersive virtual reality exercises depicting IADLs.	10 sessions, twice weekly, 45 minutes per session. Total 9-hour dosage.	Usual care	Lawton-Brody IADL scale	No statistically significant effect on functionality between pre- and post-test.
Orgeta et al. (2015)	Multi-centre, single-blind RCT	Level I	UK; *N* *=* 356 caregiving dyads, community-dwelling.	iCST	Sessions completed up to three times weekly over 25 weeks. 30 minutes per session, 75 total.	Usual care	Bristol ADL Scale	No significant between-group difference at week 26 (p = 0.36)
Orrell et al. (2014)	RCT	Level I	UK, *N* *=* 236, care home residents and community-dwelling.	MCST (previously received CST programme)	24 sessions total, 1 session weekly.	Usual care	ADCS-ADL	At 3 months, MCST group had significantly better scores than control on the ADCS-ADL (p = 0.04), no significant difference at 6 months.
Orrell et al. (2005)	Pilot RCT	Level I	UK, *N* *=* 35, recruited from four residential homes	MCST (previously completed CST programme)	Sessions completed once a week over 16 weeks	Group that previously received CST but no MCST, and group that received no CST or MCST. Both groups received usual care	CAPE-BRS	No significant difference between assessment phases of CAPE-BRS (p = 0.39).
Piras et al. (2017)	Single-blind, multi-centre RCT	Level I	Italy, *N* *=* 35, vascular dementia, residential homes	CST adapted to the Italian context	14 group sessions, twice a week for 7 weeks	Active control; educational activities involving reading stories/newspapers, discussion, creative activities	DAD	No significant between-group difference as measured by DAD
Schecker et al. (2013)	Single-blind RCT	Level I	Germany, *N* *=* 42, all outpatient participants medicated with AChEIs.	‘Focus group’ involving discussions ‘Training group’ involving cognitive-specific exercises	Not reported	Usual care	Barthel Index, NOSGER family survey on ADL/IADL	Significant results found for BI between all groups, Significant difference for IADLs between group 2 and 3, and group 1 and 3. Training group demonstrated better results for ADL/IADL than focus group.
Serdá I Ferrer and del Valle (2014)	Quasi-experimental design, pre-test and post-test	Level II	Spain, *N* *=* 64, AD, average age = 75.53 years, 39.06% severe dementia. Community dwelling participants.	Rehabilitation programme (RP): multicomponent therapy consisting of physical exercise, cognitive re-education and psychomotor stimulation	8 sessions held twice per week, 60 minutes per session	N/A	Barthel Index	No significant change in ADLs as measured by BI (p = 0.901)
Soedirman et al. (2021)	Case report	Level V	Indonesia, community-dwelling 76-year-old man with vascular dementia.	CST	Several sessions of CST received for 6 months	N/A	Not stated	Improvement in ADL abilities observed at third, sixth and ninth month.
Spector et al. (2003)	Single-blind, multi-centre RCT	Level I	England, *N* = 201, mild to moderate dementia; 18 residential homes and 5 day care centres	CST	14 sessions over 7 weeks, twice a week, 45 minutes each.	Control group received usual care	CAPE-BRS	No significant between-group difference in functional ability (p=0.449)
Tanaka et al. (2021)	Quasi-randomised controlled trial	Level II	Japan, *N* *=* 31, geriatric health service facility.	Group-based motor and cognitive combined intervention, in addition to usual care	45-minute sessions, twice weekly over 8 weeks	Usual care	Barthel Index	No significant between-group differences in ADL (p = 0.0610)
Yamagami et al. (2012)	RCT	Level I	Japan, *N* *=* 54, mean age 85.2 years, residential care homes	Brain-activating rehabilitation (reality orientation combined with creative activities and reminiscence therapy)	1-hour sessions, twice weekly for 12 weeks. Total 24 sessions.	No treatment	Multidimensional observation scale for elderly subjects (MOSES)	Intervention group had significantly lower scores for total score of MOSES (*p* = 0.048). self-care specific domain not significant (*p* = 0.186)

RCT: randomised controlled trial; CST: cognitive stimulation therapy; CS: cognitive stimulation; AChEIs: acetylcholinesterase inhibitors; ADL: activities of daily living; IADL: instrumental activities of daily living.

a Level of evidence presented using John Hopkins nursing evidence-based practice evidence level and quality guide (Dang and Dearholt, 2017).

## Results

### Search results

**Figure 1.**
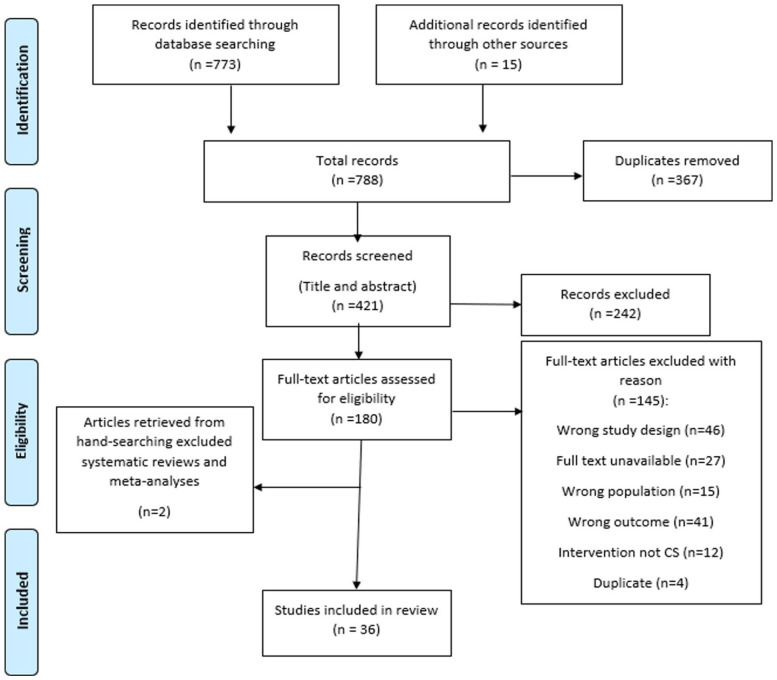
PRISMA Flow Diagram Source: Moher et al. (2009).

The three-step search strategy yielded a total of 788 records. Following removal of duplicates, the titles and abstracts of 421 articles were screened with regards to inclusion and exclusion criteria. Any records reviewers were unsure of at this phase were included for the full-text screen. One hundred eighty records were included for assessment of eligibility through a full-text screen. Systematic reviews and meta-analyses excluded at this stage were screened, yielding an additional two papers of relevance to be included. One hundred forty-five records overall were excluded after the full-text screen due to wrong study design (*n* = 46), wrong population (*n* = 15), no evaluation of ADL outcomes (*n* = 41) and no CS involved in the intervention programme (*n* = 12). An additional four papers were excluded due to being duplicates that had not been removed at an earlier screening stage. Twenty-seven papers were also excluded due to the full text being unavailable. Figure 1 depicts a PRISMA flow diagram indicating each stage of study selection.

### Characteristics of included studies

Thirty-six papers studying a total of 2495 participants from across 13 different countries were included. Study publication years ranged from 1994 to 2022. Table 3 summarises the interventions evaluated of included papers. Participant demographics varied; studies evaluated participants with a range of dementia sub-types including Alzheimer’s disease (AD), vascular dementia (VaD), Parkinson’s disease dementia (PDD), frontotemporal dementia and dementia with Lewy bodies (DLB). Three studies included both individuals with MCI and individuals with dementia in their sample (Leroi et al., 2019; Muñiz et al. 2015; Olazarán et al., 2004). All studies included evaluated the effect of CS on ADLs quantitatively, either as a primary or secondary outcome. While a formal critical appraisal of study quality was not conducted, the majority of included papers were considered to be of a high level of evidence, with 26 studies involving a level I randomised controlled trial (RCT) design. A summary of study designs of included papers can be viewed in Table 4. Studies were grouped and discussed by intervention approaches. These groupings are as follows: (1) CST programmes, (2) Group CS programmes, (3) Multi-component CS programmes, (4) Individual CS programmes and (5) other types of CS.

**Table 3.** Types of CS intervention.

Intervention type	*n (*%
Cognitive stimulation therapy	8 (22.2)
Multi-component cognitive stimulation programme	7 (19.4)
Group cognitive stimulation programme	7 (19.4)
Individual cognitive stimulation programme	4 (11.1)
Cognitive-motor stimulation	3 (8.3)
Maintenance cognitive stimulation therapy	2 (5.6)
Occupational therapy cognitive stimulation programme	2 (5.6)
Individual cognitive stimulation therapy	2 (5.6)
Computerised cognitive stimulation programme	1 (2.8)

**Table 4.** Designs of included studies.

Study Design	*n* (%)
Randomised controlled trial	22 (61.1)
Pilot randomised controlled trial	5 (13.9)
Quasi-experimental	4 (11.1)
Quasi-randomised controlled trial	1 (2.8)
Non-randomised controlled trial	1 (2.8)
Case study	1 (2.8)
Case series	1 (2.8)
Observational	1 (2.8)

### CST programmes

Eight papers included in this review evaluated an intervention of CST. Spector et al.’s (2003) RCT originally trialled the English format of CST (*N* = 201), which two smaller-scale, Irish-based trials further evaluated (Coen et al., 2011; Dolan et al., 2022). All three studies involved 45-minute group sessions run twice weekly for a duration of 7 weeks. The CST programme followed consists of reality orientation and a warm-up activity, followed by themed activities and discussions. Physical games, word association, object categorisation and number games were included, with multi-sensory stimulation incorporated where possible. Neither Spector et al. (2003) or Coen et al. (2011) found significant between-group differences in functional ability when compared to a usual-care control group. No significant between-group differences in ADL performance were also reported in Dolan et al.’s (2022) study comparing CST to Sonas.

Four RCTs evaluated a programme of CST adapted to their relevant cultures. Two studies evaluated the Italian adaptation of CST (CST-IT) (Capotosto et al., 2017; Piras et al., 2017), Alvares-Pereira et al. (2021) evaluated CST-Portuguese, and Marinho et al. (2021) evaluated CST-Brazil. All studies followed a similar programme-structure to Spector et al.’s (2003) English format, however adapted the programme to involve culturally relevant songs, foods, famous faces and games. The Italian and Portuguese studies all conducted 14, 45-minute sessions twice weekly over 7 weeks. Marinho et al.’s (2020) RCT followed a similar structure. Overall, only two studies reported favourable outcomes for functional abilities (Alvares-Pereira et al., 2021; Marinho et al., 2021). However, results from Marinho et al.’s (2021) CST-Brazil should be interpreted with caution due to the small sample size. Soedirman et al.’s (2021) case report evaluated the use of CST in a 76-year-old Indonesian man with VaD, observing an improvement in ADL performance 9 months after initiation of CST.

However, the study does not provide detail of intensity, duration, or characteristics of intervention. Furthermore, outcome measures used to evaluate ADL abilities are not clearly stated. Combined with the case report design, limited conclusions and generalisations can be drawn from this study.

Two papers evaluated the effects of MCST in participants who had previously received the standard CST programme (Orrell et al., 2005, 2014). MCST sessions follow a similar format to CST. Orrell et al.’s (2005) pilot RCT (*N* = 35) of 16-once-per-week sessions of MCST reported no significant improvement in functional abilities post-intervention. A larger-scale RCT (*N* = 236) evaluated MCST of greater duration, at 24 sessions total (Orrell et al., 2014). Significant between-group differences were observed in ADL performance at 3-months; however, this was not observed at 6 months. The demographics between Orrell et al. (2005) and (2014) differed, with community-dwelling adults included in the later study compared to only nursing home residents in the pilot study.

### CS programmes

Seven studies evaluated group CS programmes. Characteristics of the CS programmes and participant demographics differed across studies. Two studies evaluated a CS program consisting of reality orientation, discussion and reminiscence (Chapman et al., 2004; Yamagami et al., 2012). Chapman et al. (2004) conducted once-weekly, 5-hour sessions for 8 weeks. 41 community dwelling adults with AD receiving donepezil were recruited. Yamagami et al. (2012) delivered a total of 24 1-hour sessions, twice weekly for 12 weeks with institutionalised participants (*N* = 54). The more recent study of Alves et al. (2014) reported a similar structure to CS, however incorporated activities around object identification and categorisation, memory exercises and sequencing and planning of tasks. 1-hour sessions were conducted at a greater frequency than the previous two studies, at three times per week. However, CS was not reported to have any significant effect on ADLs in any of the three studies.

Farina et al. (2006) and Schecker et al. (2013) both compared two different modalities of cognitive intervention. Schecker et al.’s (2013) RCT compared two different modalities of cognitive stimulation (CS), through a ‘training group’ geared at working memory and executive processes, and a discussion-based ‘focus group’. Farina et al.’s (2006) quasi-experimental study took a similar approach, comparing a ‘global stimulation’ group with a cognitive-oriented training group. While detail of intervention sessions was not reported by Schecker et al. (2013), Farina et al.’s (2006) global stimulation group consisted of conversation, singing, dancing and games. Fifteen sessions were conducted over 6 weeks, with 3-hours allocated per session. Both global stimulation groups from these studies demonstrated an improvement in ADL/IADL performance. However, Schecker et al.’s (2013) cognitive training group demonstrated more significant improvements when compared to the ‘focus group’, while Farina et al.’s (2006) ADL improvements were lost at follow-up.

Breuil et al.’s (1994) RCT was the oldest study included in this review. Their CS program involving pen-and-paper cognitive exercises, discussion, identification and categorisation activities found no effect on ADL outcomes. However, Breuil et al. (1994) reported the outcome measure used was not suitable for individuals with mild-to-moderate dementia. Furthermore, Folkerts et al.’s (2018) adaptation of ‘NEUROvitalis senseful’, a structured CS program adapted for older adults with PDD, found significant deterioration in ADL performance as measured by the Barthel Index. However, this was attributed to the nature of the condition.

### Multicomponent CS programmes

Overall, nine included papers evaluated CS as part of a wider multi-component approach to intervention. Three studies evaluated MAKS, a manualised, group therapy consisting of tasks organised into three categories: motor stimulation, ADLs and CS (Graessel et al., 2011; Luttenberger et al. 2012a, 2012b). Each MAKS session consists of an introductory song or discussion, followed by physical activities and games, individual/group cognitive exercises and participation in ADL or IADL, such as preparing a snack or gardening. MAKS sessions lasted overall 2-hours and were completed 6 days a week. Graessel et al.’s (2011) RCT (*N* = 61) reported ADL abilities of MAKS group remained stable at 12 months compared to a deterioration of the treatment-as-usual control group. Luttenberger et al.’s (2012b) RCT (*N* = 52) demonstrated similar results, however ADL abilities of the MAKS group deteriorated significantly between end of therapy and follow-up. The larger-scale RCT (*N* = 139) noted a significant difference in ADL abilities of MAKS group after 6 months of intervention (Luttenberger et al., 2012a). All three studies were conducted in nursing homes.

Serdá i Ferrer and del Valle (2014) evaluated the effect of a rehabilitation programme (RP) on 64 Spanish adults with AD through a quasi-experimental design. Eight, 1-hour sessions were held twice weekly. Sessions began with a reality orientation and warm-up, followed by either exercise or a recreational activity like gardening, supplemented with cognitive exercises. The session was concluded with multi-sensory relaxation strategies. Maci et al.’s (2012) pilot study followed a similar approach to intervention, combining physical activity, CS and socialisation. This approach was more intensive than the RP, delivering therapy 5 days a week for 3 months, with 3-hours per session. CS activities included real-life problem solving, puzzles, money tasks, reminiscence and planning daily activities. Neither study demonstrated a significant effect on ADL performance. However, 39.06% of participants recruited for Serdá i Ferrer and del Valle’s (2014) study were diagnosed with severe dementia which contributed to attrition and interfered with outcome measurement.

Two studies implemented multi-component CS programmes in day-care centres. Ibarria et al. (2016) evaluated an integrated psycho-stimulation programme (IPP) designed to enhance ADL independence. Two hundred six participants received a daily dose of AChEIs combined with IPP delivered 5 days a week, from 10 am to 6 pm. The programme consisted of three workshops: a cognitive workshop, a psycho-expression workshop and an occupational workshop. Chang et al. (2021) carried out a smaller-scale study (*N* = 23) of a multi-component CS programme (MCCSP). The MCCSP contained music-leading exercise activities followed by structured CS activities. CS activities included singing, playing musical instruments, board games and educational classes. The MCCSP was delivered for 1-hour sessions, three times a week for 12 weeks. ADL outcomes from both studies were not promising; worsening on ADL functionality was observed in the IPP group, however results were not clinically significant. Furthermore, not all participants were subjected to the same treatment duration, impacting results. Chang et al. (2021) also found no significant post-treatment effect on functional ability.

Two articles studied an occupational therapy-based CS intervention (Ávila et al., 2018; Jiménez-Palomares et al., 2021). A double-blind RCT (*N* = 58) evaluated the effect of an occupational therapy programme based on the training of ADL through CS (Jiménez-Palomares et al., 2021). Standard approaches to CS including reality orientation and discussion were combined with ADL-focused identification and categorisation activities. Sessions were conducted for 5 weeks. While CS was central to Jiménez-Palomares et al.’s (2021) study, Ávila et al. (2018) incorporated CS into a wider multi-component occupational therapy approach consisting of CS, meaningful activities, psychomotor and sensory skills activation, caregiver counselling and ADL training. Ninety-minute sessions were delivered for 12 weeks, twice weekly, with an additional 8 weeks of sessions implemented after a 1.5-month intervention withdrawal. Both demonstrated statistically significant changes in ADLs post-intervention, however these results were not maintained at follow-up for Jiménez-Palomares et al. (2021) when compared to a conventional occupational therapy programme. Furthermore, attrition was noted as a limitation to this study, impacting the results at follow-up.

### Individual CS programmes

A total of six articles evaluated programmes of individual CS. Cruz et al.’s (2015) case report conducted 1.5-hour weekly implementation sessions with the caregivers of five Brazilian adults with AD. CS activities consisted of reality orientation and object identification. Recreational activities like knitting, sport, board games and reading were also included. D’onofrio et al.’s (2015) pilot RCT (*N* = 90) evaluated the effect of a rivastigmine transdermal patch (RTP; 9.5 mg/24 hours dose) combined with individual CS. The CS programme was adapted for cognitive level, with 90-minute sessions delivered once a week for two cycles of 2 months. Sessions focused on the training of orientation, social skills, memory, attention, logic and verbal fluency. D’onofrio et al.’s (2016) smaller-scale RCT (*N* = 40) repeated this programme for four cycles. Cruz et al. (2015) reported an improvement of IADL score for three participants. However, conclusions are limited due to the case report design. Significant between-group differences were observed in ADLs and IADLs for the RTP + CS group when compared to an RTP only control group (D’Onofrio et al., 2015). Significant differences were also demonstrated in IADLs after 1 year at follow-up for D’Onofrio et al.’s (2016) CS group. However, both studies are limited by their relatively small sample size.

A large-scale, multi-centre RCT was conducted with 356 caregiving dyads to evaluate the clinical effectiveness of iCST (Orgeta et al., 2015). Thirty-minute sessions were completed up to three times weekly by care-partners, over 25 weeks. Sessions involved various themes and activities, including discussion, object categorisation, quizzes, number games, creative activities and physical games. Leroi et al. (2019) adapted this iCST programme for use with PDD, DLB and PD-MCI, in a programme named PD-CST. Similar sessions to Orgeta et al. (2015) were conducted over 12 weeks. Justo-Henriques et al.’s (2022) RCT (*N* = 59) involved a similar home-based, individual CS programme (iCS) delivered by a therapist for 45-minutes, weekly, for a total of 47 sessions. When compared to a usual care control group, no significant between-group difference was found in functional ability, however a group × time significant interaction was found for the iCS group. No significant difference was found for the PD-CST or iCST group in functional ability. However, results of Leroi et al.’s (2019) PD-CST study must be interpreted with caution due to the sample including participants with MCI. Furthermore, ADL performance was a secondary outcome for all three studies.

### Other CS interventions

The remaining four articles involved cognitive-motor stimulation (Muñiz et al., 2015; Olazarán et al., 2004; Tanaka et al., 2021), and a computerised CS programme (Oliveira et al., 2021).

The effects of a cognitive-motor stimulation intervention (CMSI) were evaluated at 12 months by Olazarán et al. (2004) and at 3 years by Muñiz et al. (2015). A total of 84 participants with MCI and mild-to-moderate AD were included in both RCTs. The CMSI programme consisted of 3.5-hour sessions, twice weekly. Sessions consisted of individual and group cognitive exercises, reality orientation, psychomotor therapy, and ADL training. At 12 months, Olazarán et al. (2004) found no significant effect of intervention on functional abilities. At 3 years, Muñiz et al. (2015) reported both CMSI and the control group showed progressive deterioration in functional abilities, however ADL and IADL results were more favourable for the CMSI group. Tanaka et al. (2021) took a similar approach to intervention, with cognitive training or stimulation activities preceded by aerobic exercise or stretching. Group-based sessions lasted 45-minutes and took place twice weekly for 8 weeks in addition to usual care. However no significant improvements in ADL were reported.

Oliveira et al.’s (2021) computerised CS programme consisted of non-immersive virtual reality exercises depicting several IADLs, including grocery shopping and kitchen tasks. The programme consisted of 12, 45-minute CS sessions distributed over 2 days a week. Despite the intervention being delivered through the medium of IADLS, functional performance was a secondary outcome, with no significant effect on functionality reported.

## Discussion and implications of findings

The aim of this scoping review was to chart the evidence available for the use of CS in improving ADL outcomes. Approaches to CS varied across the studies retrieved; CS was delivered through individual or group formats, through manualised therapy approaches, via virtual reality, or as part of a wider, multi-component programme. While a formal quality appraisal was not conducted due to the nature of scoping reviews, studies conducted on this topic generally appeared to be of a high level of evidence with a total of 27 RCTS. Multi-component CS interventions were the most common form of CS programme reviewed, with a total of nine papers included. This multi-component approach yielded the most promising outcomes for ADLs, as seen through the MAKS and CMSI programmes. Both programmes consisted of typical CS activities like discussion and reality orientation, incorporated with motor activities, ADL training and cognitive exercises. Two occupational therapy-based multi-component interventions also yielded promising results for ADL outcomes, through the incorporation of ADL-focused tasks into a typical CS programme (Jiménez-Palomares et al., 2021), or the provision of CS and ADL training as part of a broader occupational therapy intervention (Ávila et al., 2018).

The outcomes of these multi-faceted interventions are promising when compared to traditional CST or CS programmes. Six of the eight CST studies reviewed reported no significant effect on ADL performance, and of the seven group CS programmes reviewed, only one study reported improvements in ADL and IADL performance (Farina et al., 2016). Overall results of individual CS and MCST were not promising for ADLs. The majority of these programmes followed a similar approach, with reality orientation, discussion and reminiscence being core intervention components. While this traditional approach to CS has been extensively supported for benefiting cognition (Woods et al., 2012), these changes in cognition do not appear to transfer to ADL performance, a phenomenon previously noted by Bherer (2015). This overall lack of evidence supporting CS programmes for improving ADL outcomes has been reflected in previous systematic reviews (Aguirre et al., 2013; Woods et al., 2012). However, ADLs were not primary outcomes for multiple CS studies reviewed, potentially explaining the overall lack of benefit for ADLs reported, as Spector et al. (2003) suggests secondary outcome measures used may not be sensitive enough to detect the impact of CS on functional abilities. Findings from Breuil et al. (1994) consolidate this point, as the authors noted the outcome measure used was not designed for their sample population, invalidating results.

While the findings of multi-component interventions are promising, several limitations exist. MAKS and CMSI are both intensive programmes in comparison to standard CST. While the greater intensity of these programmes may contribute to their overall benefit, all three MAKS studies and Muñiz et al.’s (2015) CMSI study recruited institutionalised participants with dementia. Residential settings may facilitate a more intensive programme than a community setting. However, the majority of people with dementia in Ireland live in the community (Connolly et al., 2014). The application of these studies to community settings may therefore be limited, due to the limited time and resources available for Irish community occupational therapists (McGrath et al., 2014). While Ávila et al.’s (2018) occupational therapy programme and Olazarán et al.’s (2004) implementation of CMSI with community-dwelling adults demonstrated benefits for ADLs, further studies are needed to evaluate the feasibility and effectiveness of community-based, multi-component CS interventions. Therefore, the results of this review will align with the results of a pilot CS ADL group currently underway to inform a research proposal for a larger trial of a community-based, multi-component CS group for people with mild-to-moderate dementia aimed at improving ADL outcomes.

## Limitations of this review

Despite efforts taken to ensure rigour by following the framework of Arksey and O’Malley (2005) as extended upon by Levac et al. (2010) and Peters et al. (2020), the limitations of this review cannot be overlooked. Important studies may have been missed during the search process, due to the exclusion of non-English language articles and inaccessible articles behind a paywall. Studies evaluating participants with dual diagnoses were also excluded. Furthermore, the quality and risk of bias of studies included was not assessed, therefore limited conclusions and generalisations can be drawn from this review. However, this is a limitation of the methodology itself as scoping reviews do not aim to critically appraise evidence included (Aromataris and Munn, 2020). A systematic review should therefore be conducted in future to assess the certainty of evidence available. Furthermore, while best practice reporting criteria as per the PRISMA-ScR guidelines were mainly followed, a protocol for this review was not registered or published. This limits the reliability and reproducibility of this review. Only quantitative papers were retrieved during the search process. This review was therefore limited by the lack of rich experiential information qualitative research could provide regarding the use of CS in ADLs. Future studies should evaluate the qualitative perspective of participants engaging in CS ADL interventions in order to inform future programmes.

## Conclusion

This scoping review aims to inform future CS studies aiming to improve ADL outcomes for adults with mild-to-moderate dementia. Findings from this review chart a range of CS programmes from across 13 countries. The majority of studies were indicated to be of a high level of evidence, with multi-component CS programmes involving ADL-based activities reporting significantly greater impact on ADLs than standard CS or CST. Group CS programmes were also indicated to have more benefit than individual CS. It is therefore suggested that to achieve improvements in ADLs, future CS programmes should incorporate ADLs into group intervention. Multi-component CS interventions that demonstrated significant benefits for ADLs however reported more intensive interventions, which may act as a barrier to future implementation of these programmes outside of residential settings. Future studies should consider the feasibility and effectiveness of multi-component CS intervention implementation in community settings.

**Key findings**

- Multi-component CS interventions demonstrated greater benefits for ADLs in comparison to other forms of CS.
- Incorporation of ADL-focused activities into intervention benefits functional outcomes.
- Traditional CS does not benefit ADL outcomes for individuals with dementia.

**What the study has added**

This review informs occupational therapists planning to deliver CS within their practice. This review has also analysed gaps in the literature, calling for future qualitative research, feasibility, and efficacy studies of community-based CS ADL programmes.

## Appendix

**Appendix A1.** Full search strategy per database.

Database	Search strategy	Papers retrieved
MEDLINE	exp Dementia/ limit 1 to (full text and english language) (dementia or alzheimer disease or alzheimer* or lewy body disease or frontotemporal dementia or vascular dementia or mixed dementia or major neurocognitive disorder).mp. [mp=title, abstract, original title, name of substance word, subject heading word, floating sub-heading word, keyword heading word, organism supplementary concept word, protocol supplementary concept word, rare disease supplementary concept word, unique identifier, synonyms] limit 3 to (full text and English language) 2 or 4 “Activities of Daily Living”/ limit 6 to (full text and English language) (activities of daily living or adl or functional status or functionality or everyday function*).mp. [mp=title, abstract, original title, name of substance word, subject heading word, floating sub-heading word, keyword heading word, organism supplementary concept word, protocol supplementary concept word, rare disease supplementary concept word, unique identifier, synonyms] limit 7 to (full text and English language) 7 or 9 (“cognitive stimulation” or “cognitive stimulation therapy” or cst or cs).mp. [mp=title, abstract, original title, name of substance word, subject heading word, floating sub-heading word, keyword heading word, organism supplementary concept word, protocol supplementary concept word, rare disease supplementary concept word, unique identifier, synonyms] limit 10 to (full text and English language) 5 and 10 and 12	62
CINAHL	( dementia or alzheimer disease or alzheimer* or vascular dementia or mixed dementia or frontotemporal dementia or major neurocognitive disorder ) AND ( activities of daily living or adl or functional status or functionality or everyday function* ) AND ( cognitive stimulation therapy or cst or cs or cognitive stimulation )	20
PubMed	( ((dementia[Title/Abstract] OR “vascular dementia”[Title/Abstract] OR “frontotemporal dementia”[Title/Abstract] OR alzheimer*[Title/Abstract] OR “alzheimer disease”[Title/Abstract] OR “major neurocognitive disorder”[Title/Abstract] OR “lewy body disease”[Title/Abstract] OR “mixed dementia”[Title/Abstract]) OR (dementia[MeSH Terms])) AND ((activities of daily living[MeSH Terms]) OR (“activities of daily living”[Title/Abstract] OR adl[Title/Abstract] OR “functional status”[Title/Abstract] OR functionality[Title/Abstract] OR “everyday function*”[Title/Abstract])) AND (“cognitive stimulation therapy”[Title/Abstract] OR “cognitive stimulation”[Title/Abstract] OR cst[Title/Abstract] OR cs[Title/Abstract]) ) Filters: Full text, English	86
Cochrane	#1 (“cognitive stimulation” OR “cognitive stimulation therapy” OR CST OR CS):ti,ab,kw (Word variations have been searched) #2 MeSH descriptor: [Activities of Daily Living] this term only #3 (“activities of daily living” OR adl OR “functional status” OR (everyday NEXT function*) OR functionality):ti,ab,kw (Word variations have been searched) #4 MeSH descriptor: [Dementia] explode all trees #5 (dementia OR “alzheimer disease” OR alzheimer* OR “vascular dementia” OR “frontotemporal dementia” OR “mixed dementia” OR “lewy body disease” OR “major neurocognitive disorder”):ti,ab,kw (Word variations have been searched) #6 #2 OR #3 #7 #4 OR #5 #8 #1 AND #6 AND #7	214
Scopus	(TITLE-ABS-KEY ( dementia OR “vascular dementia” OR “mixed dementia” OR “frontotemporal dementia” OR “lewy body disease” OR alzheimer* OR “major neurocognitive disorder” OR “alzheimer disease” ) AND TITLE-ABS-KEY ( “cognitive stimulation” OR “cognitive stimulation therapy” OR cst OR cs ) AND TITLE-ABS-KEY ( “activities of daily living” OR adl OR functionality OR “functional status” OR “everyday function*” ) ) AND ( LIMIT-TO ( LANGUAGE, “English” ) )	118
PsycINFO	exp Dementia/ limit 1 to (full text and english language) (dementia or alzheimer disease or alzheimer* or vascular dementia or lewy body disease or mixed dementia or frontotemporal dementia or major neurocognitive disorder).mp. [mp=title, abstract, heading word, table of contents, key concepts, original title, tests & measures, mesh word] limit 3 to (full text and english language) “Activities of Daily Living”/ limit 5 to (full text and english language) (“activities of daily living” or adl or functionality or functional status or everyday function*).mp. [mp=title, abstract, heading word, table of contents, key concepts, original title, tests & measures, mesh word] limit 7 to (full text and english language) Cognitive Stimulation Therapy/ limit 9 to (full text and english language) (“cognitive stimulation therapy” or “cognitive stimulation” or cst or cs).mp. [mp=title, abstract, heading word, table of contents, key concepts, original title, tests & measures, mesh word] limit 11 to (full text and english language) 2 or 4 6 or 8 10 or 12 13 and 14 and 15	57
OTseeker	[Any Field] like ‘“cognitive stimulation” OR “cognitive stimulation therapy” OR cst OR cs’ AND [Any Field] like ‘dementia or “alzheimer disease” OR alzheimer* OR “mixed dementia” OR “vascular dementia” OR “frontotemporal dementia” OR “major neurocognitive disorder” OR “lewy body disease”‘ AND [Any Field] like ‘“activities of daily living” OR adl OR “functional status” OR “functionality” OR “everyday function*”‘	2
Embase	(‘dementia’/exp OR ‘dementia’ OR ‘alzheimer disease’ OR ‘vascular dementia’ OR ‘mixed dementia’ OR ‘frontotemporal dementia’ OR ‘lewy body disease’ OR ‘major neurocognitive disorder’ OR ‘alzheimer*’) AND (‘cognitive stimulation therapy’ OR ‘cognitive stimulation’ OR cst OR cs) AND (‘daily life activity’ OR ‘activities of daily living’ OR adl OR functionality OR ‘functional status’ OR ‘everyday function*’) AND [english]/lim	214
